# *N*-Alkyl-Substituted Isatins Enhance P2X7 Receptor-Induced Interleukin-1*β* Release from Murine Macrophages

**DOI:** 10.1155/2016/2097219

**Published:** 2016-07-21

**Authors:** Ronald Sluyter, Kara L. Vine

**Affiliations:** ^1^School of Biological Sciences, University of Wollongong, Wollongong, NSW 2522, Australia; ^2^Centre for Medical and Molecular Bioscience, University of Wollongong, Wollongong, NSW 2522, Australia; ^3^Illawarra Health and Medical Research Institute, Wollongong, NSW 2522, Australia

## Abstract

Extracellular adenosine 5′-triphosphate (ATP) activates the P2X7 receptor channel to induce the rapid release of the proinflammatory cytokine, interleukin- (IL-) 1*β*, from macrophages. Microtubule rearrangements are thought to be involved in this process. Some isatin derivatives alter microtubules and display anticancer activities. The current study investigated the effect of isatin and seven structurally diverse isatin derivatives on P2X7-mediated IL-1*β* release from murine J774 macrophages. ATP-induced IL-1*β* and lactate dehydrogenase (LDH) release were assessed by specific colorimetric assays. P2X7 activity was determined by flow cytometric measurements of ATP-induced cation dye uptake. Cytotoxicity of isatin derivatives was determined using a tetrazolium-based colorimetric assay. ATP caused rapid IL-1*β* release in a concentration-dependent manner, and this process was completely impaired by the P2X7 antagonist, AZ10606120. In contrast, 5,7-dibromo-*N*-(*p*-methoxybenzyl)isatin (NAI) and 3-{4-[5,7-dibromo-1-(4-methoxybenzyl)-2-oxoindolin-3-ylidenamino]phenyl}propanoic acid (NAI-imine) enhanced P2X7-induced IL-1*β* release by twofold compared to that of isatin and the parent molecule, 5,7-dibromoisatin. NAI and NAI-imine had minimal effect on P2X7-induced dye uptake and LDH release. In contrast, 24-hour incubation with NAI and NAI-imine (in the absence of exogenous ATP) induced macrophage death in a concentration-dependent manner. In conclusion, this study demonstrates that* N*-alkyl-substituted isatins enhance P2X7 receptor-induced IL-1*β* release from murine macrophages. Thus, in addition to direct anticancer effects, these compounds may also impact inflammatory and immune cells within the tumor microenvironment.

## 1. Introduction

The P2X7 receptor is a trimeric ligand-gated cation channel, which upon activation by its natural ligand, extracellular adenosine 5′-triphosphate (ATP), results in the rapid flux of cations including fluorescent dyes such as ethidium^+^ [1]. P2X7 is highly expressed on monocytes and macrophages and has central roles in inflammation and immunity [2, 3], largely in part, through the release of the proinflammatory cytokine, interleukin- (IL-) 1*β* [4]. P2X7 activation can induce IL-1*β* release by a number of nonclassical secretion pathways involving secretory lysosomes, microvesicles, exosomes, autophagosomes, and cell death [5]. However, P2X7-induced IL-1*β* release can occur prior to [6] or in the absence of cell death [7]. At a molecular level, microtubule rearrangements are involved in P2X7-induced IL-1*β* release. The microtubule destabilizer, colchicine, can impair P2X7-induced IL-1*β* release from murine microglia [8] and macrophages [9]. Moreover, the microtubule disrupting agent, nocodazole, as well as the histone deacetylase inhibitors, suberoylanilide hydroxamic acid and ITF2357 (which impair tubulin hyperacetylation), can prevent P2X7-induced IL-1*β* release from human monocytes [10]. In contrast, the microtubule stabilizing agent, paclitaxel (Taxol), augments P2X7-induced IL-1*β* release from these cells [10].

Isatin (1*H*-ondole-2,3-dione) is an endogenous molecule found in plants and animals including humans and other mammals [11]. In the tissues of mammals, isatin concentrations vary from <0.1 to 10 *μ*M [11]. Due to the synthetic versatility of isatin, modification of this compound has resulted in the generation of a large number of isatin derivatives, which demonstrate a diverse array of antimicrobial, anti-inflammatory, anticonvulsant, and anticancer properties [12–14]. The biological targets of isatin derivatives are varied and include proteases, kinases, and caspases [15]. Moreover, Vine and colleagues have generated a number of* N*-alkylated isatin derivatives with potent cytotoxic activity against tumor cells, which act by binding to tubulin and destabilizing microtubules [16]. Therefore, given the potential role of the microtubule network in P2X7-mediated IL-1*β* release, the current study investigated the effect of a panel of isatin derivatives on P2X7-mediated IL-1*β* release from murine J774 macrophages.

## 2. Material and Methods

### 2.1. Materials

RPMI-1640 medium and GlutaMAX were from Life Technologies (Grand Island, USA). Fetal calf serum was from Bovogen Biologicals (Keilor East, Australia). Lipopolysaccharide (LPS) (*Escherichia coli* serotype 055:B5) and ATP were from Sigma Chemical Co. (St. Louis, USA).* N*-[2-[[2-[(2-Hydroxyethyl)amino]ethyl]amino]-5-quinolinyl]-2-tricyclo[3.3.1.13,7]dec-1-ylacetamide dihydrochloride (AZ10606120) was from Tocris Bioscience (Ellisville, USA). Isatin and 5-bromoisatin were from Aldrich Chemical Co. (Milwaukee, USA). 5-Fluoroisatin was from Alfa Aesar Co. (Ward Hill, USA). 5,7-Dibromoisatin, 5-nitroisatin, and 1*N*-methylisatin were prepared as described in [17]. 5,7-Dibromo-*N*-(*p*-methoxybenzyl)isatin (NAI) was prepared as described in [18]. 3-{4-[5,7-Dibromo-1-(4-methoxybenzyl)-2-oxoindolin-3-ylidenamino]phenyl}propanoic acid (NAI-imine) was prepared as described in [19, 20]. Dimethyl sulfoxide (DMSO) and ethidium bromide were from Amresco (Solon, USA). CellTiter 96® AQ_UEOUS_ One Solution was from Promega Corporation (Madison, USA).

### 2.2. Cells

The murine macrophage cell line, J774 (American Type Culture Collection, Rockville, USA), was maintained in complete medium (RPMI-1640 medium containing 2 mM GlutaMAX and 10% heat-inactivated fetal calf serum) at 37°C/5% CO_2_.

### 2.3. IL-1*β* and Lactate Dehydrogenase Release Assays

Cells in complete medium in 24-well plates (5 × 10^5^ cells/1 mL/well) were incubated overnight at 37°C/5% CO_2_. The medium was removed and the cells incubated for 4 h with 1 *μ*g/mL LPS in complete medium (1 mL/well). Cells were then washed three times with physiological medium (147 mM NaCl, 2 mM KCl, 2 mM CaCl_2_, 1 mM MgCl_2_, and 10 mM HEPES; pH 7.4). Cells were finally preincubated for 30 min in the absence or presence of compound (as indicated) in physiological medium (0.5 mL/well) and then incubated for 20 min in the absence or presence of ATP (as indicated). The samples were centrifuged (11,000 ×g for 30 s) and the cell-free supernatants were stored at −80°C until required. IL-1*β* concentration was measured using the Mouse IL-1*β* ELISA MAX*™* Deluxe Set (BioLegend, San Diego, USA) according to the manufacturer's instructions. Lactate dehydrogenase (LDH) activity in cell-free supernatants and cell lysates was measured using the Cytotoxicity Detection Kit^PLUS^ (Roche, Mannheim, Germany) according to the manufacturer's instructions. LDH release is presented as a percentage of maximal LDH release.

### 2.4. Ethidium^+^ Uptake Assay

Cells in flasks, cultured to an equivalent cell density to that of cells in the IL-1*β*/LDH release assays, were incubated for 4 h with 1 *μ*g/mL LPS in complete medium. Cells were harvested by mechanical scraping and washed three times with physiological medium. ATP-induced ethidium^+^ uptake into cells suspended in physiological medium at 37°C was determined using a flow cytometric assay as described in [21].

### 2.5. Cytotoxicity Assay

Experiments were performed as described in [22] with the following modifications. Cells in complete medium in 96-well flat bottom plates (5 × 10^3^ cells/100 *μ*L/well) were incubated overnight at 37°C/5% CO_2_. The medium was removed and the cells incubated for 4 h with 1 *μ*g/mL LPS in complete medium (100 *μ*L/well). Cells were washed once with RPMI-1640 medium (200 *μ*L/well) and then incubated with compounds (as indicated) for 24 h at 37°C/5% CO_2_ (100 *μ*L/well). Finally, cells were incubated with CellTiter 96® AQ_UEOUS_ One Solution (20 *μ*L/well) for 2 h at 37°C/5% CO_2_ and the absorbance was measured at 490 nm.

### 2.6. Data Presentation and Statistics

Data are presented as mean ± SD. Differences between treatments were compared by ANOVA (using Tukey's posttest) using Prism 5 for Mac OS X (Version 5.0a; GraphPad Software, San Diego, USA). Concentration response curves were fitted to a sigmoidal dose response using the least squares (ordinary) fit method of Prism 5 for Mac OS X.

## 3. Results

### 3.1. P2X7 Activation Induces IL-1*β* Release

**Figure 1:**
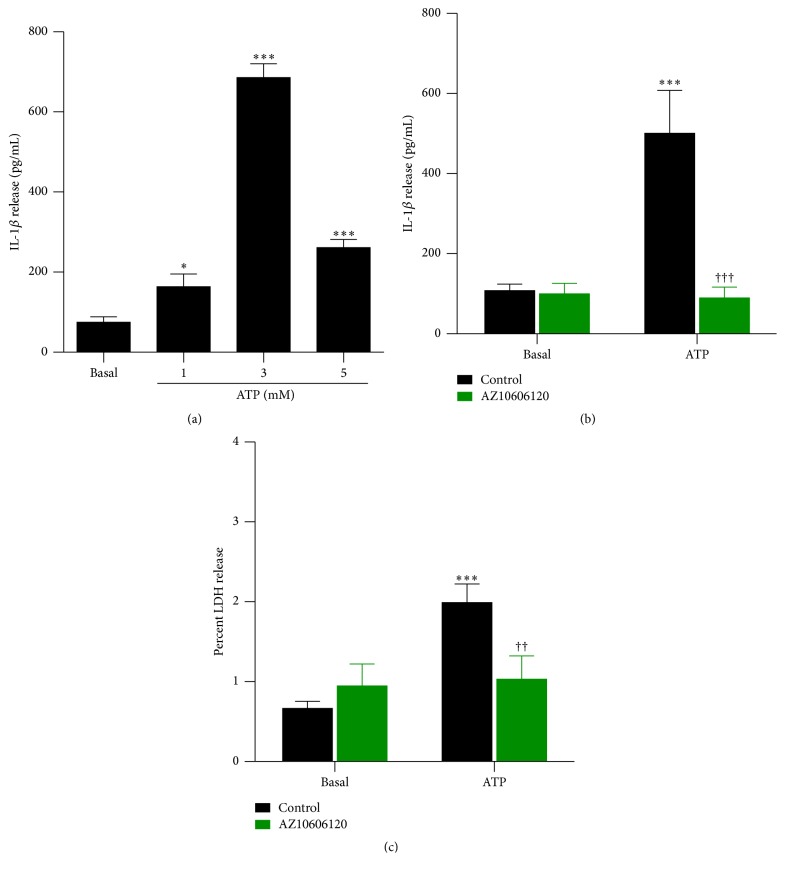
P2X7 activation induces IL-1*β* release. LPS-primed J774 cells in physiological medium were preincubated in the absence or presence of 10 *μ*M AZ10606120 for 30 min and then in the absence (basal) or presence of (a) 1, 3, or 5 mM ATP or ((b) and (c)) 3 mM ATP for 20 min. ((a) and (b)) The IL-1*β* concentration in cell-free supernatants was measured using an ELISA. (c) The LDH activity in cell-free supernatants and cell lysates was measured using a cytotoxicity kit, and results are expressed as a percentage of maximal LDH release from lysed cells. ((a) to (c)) Results are mean ± SD (*n* = 3); ^*∗*^ *P* < 0.05 or ^*∗∗∗*^ *P* < 0.001 compared with corresponding basal; ^††^ *P* < 0.01 or ^†††^ *P* < 0.001 compared with ATP alone.

P2X7 activation can induce IL-1*β* release from the J774 macrophage cell line following priming with LPS [23, 24]. To confirm these previous observations, LPS-primed J774 cells were incubated in the absence or presence of 1, 3, or 5 mM ATP, and the amount of IL-1*β* release was measured by ELISA. At each concentration examined, ATP induced significant IL-1*β* release from cells compared to cells incubated in the absence of ATP (Figure 1(a)). IL-1*β* release was maximal at 3 mM ATP (Figure 1(a)) and thus was used for subsequent studies.

To confirm further that P2X7 activation induces IL-1*β* release from J774 cells, LPS-primed J774 cells were preincubated in the absence or presence of the specific P2X7 antagonist, AZ10606120 (10 *μ*M) [25], prior to incubation with 3 mM ATP. ATP induced significant IL-1*β* release from cells compared to cells incubated in the absence of ATP (Figure 1(b)). Preincubation with AZ10606120 completely impaired the ATP-induced IL-1*β* release from these cells (Figure 1(b)). Basal IL-1*β* release in the absence or presence of AZ10606120 was similar (Figure 1(b)). ATP induced a small but significant amount of LDH release from cells (average ATP-induced LDH release of 1.32%) compared to cells incubated in the absence of ATP (Figure 1(c)). This ATP-induced LDH release was near completely prevented by preincubation with AZ10606120 (Figure 1(c)). Basal LDH release in the absence or presence of AZ10606120 was similar (Figure 1(c)). Thus, P2X7 activation induces IL-1*β* release from LPS-primed J774 cells, which may in part be a result of cell lysis.

### 3.2. NAI and NAI-Imine Increase P2X7-Mediated IL-1*β* Release in a Concentration-Dependent Manner

**Figure 2:**
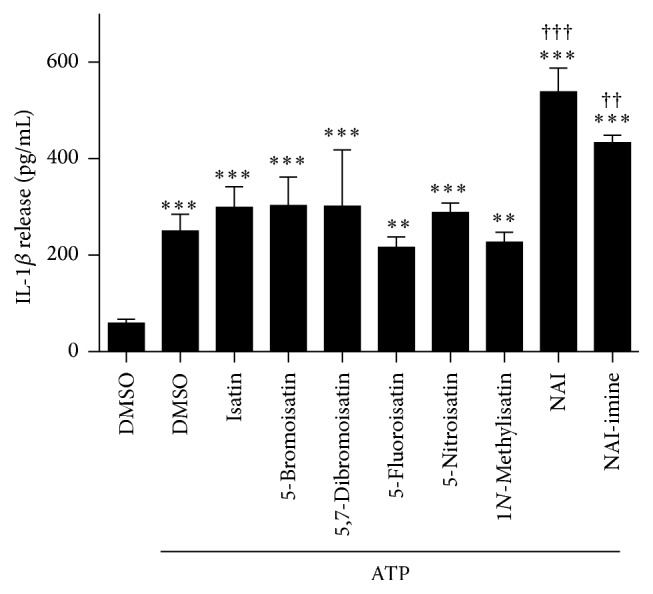
NAI or NAI-imine increase P2X7-mediated IL-1*β* release. LPS-primed J774 cells in physiological medium were preincubated in the presence of DMSO, or 1 *μ*M isatin or isatin derivative (as indicated) for 30 min, and then in the absence or presence of 3 mM ATP for 20 min. The IL-1*β* concentration in cell-free supernatants was measured using an ELISA. Results are mean ± SD (*n* = 3-4); ^*∗∗*^ *P* < 0.01 or ^*∗∗∗*^ *P* < 0.001 compared with DMSO alone; ^††^ *P* < 0.01 or ^†††^ *P* < 0.001 compared with DMSO and ATP.

To determine if isatin or isatin derivatives modulate P2X7-mediated IL-1*β* release, LPS-primed J774 cells were preincubated in the presence of DMSO (diluent control), isatin, or various isatin derivatives (each at 1 *μ*M) prior to incubation with 3 mM ATP. ATP induced significant IL-1*β* release from cells compared to cells incubated in the presence of DMSO alone (Figure 2). Preincubation with isatin or five isatin derivatives failed to modulate ATP-induced IL-1*β* release (Figure 2). In contrast, the* N*-alkylated isatin derivatives, NAI and NAI-imine, increased ATP-induced IL-1*β* release by approximately twofold that of DMSO, isatin, and the other isatin derivatives including the parent molecule, 5,7-dibromoisatin (Figure 2).

**Figure 3:**
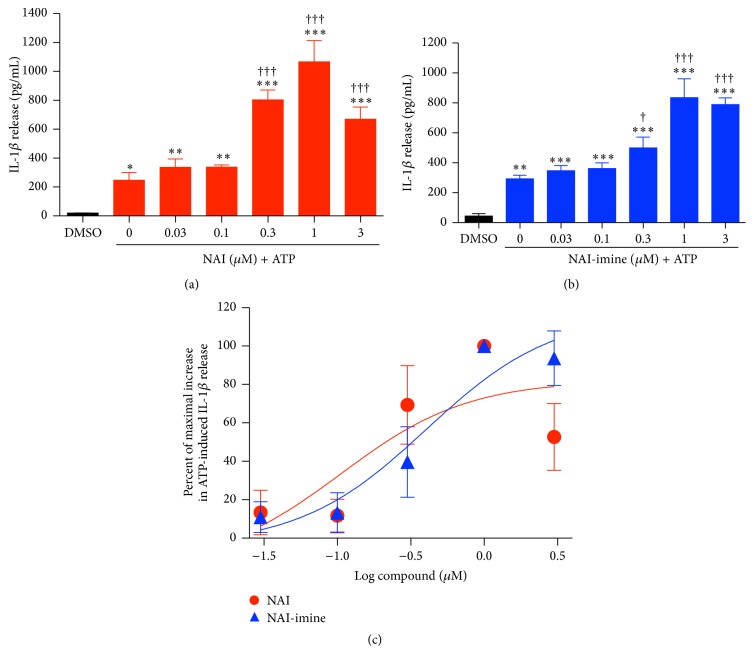
NAI or NAI-imine increase P2X7-mediated IL-1*β* release in a concentration-dependent manner. LPS-primed J774 cells in physiological medium were preincubated in the presence of ((a) and (b)) DMSO or (a) NAI or (b) NAI-imine (as indicated) for 30 min ((a) and (b)) and then in the absence or presence of 3 mM ATP for 20 min. The IL-1*β* concentration in cell-free supernatants was measured using an ELISA. (c) The percentage of maximal increase in ATP-induced IL-1*β* release was determined from (a) and (b). ((a) to (c)) Results are mean ± SD (*n* = 3); ^*∗*^ *P* < 0.05, ^*∗∗*^ *P* < 0.01, or ^*∗∗∗*^ *P* < 0.001 compared with DMSO alone; ^†^ *P* < 0.05 and ^†††^ *P* < 0.001 compared with DMSO and ATP.

To further assess if the NAI and NAI-imine effect was concentration dependent, LPS-primed J774 cells were preincubated in the presence of increasing concentrations of these two isatin derivatives prior to incubation with 3 mM ATP. Both NAI and NAI-imine increased ATP-induced IL-1*β* release in a concentration-dependent manner with maximum release observed at 1 *μ*M for both compounds (Figures 3(a) and 3(b)) and with EC_50_ values of 106 ± 12 nM and 407 ± 112 nM, respectively (Figure 3(c)).

### 3.3. NAI and NAI-Imine Have Minimal Effect on P2X7-Mediated LDH Release and Ethidium^+^ Uptake

**Figure 4:**
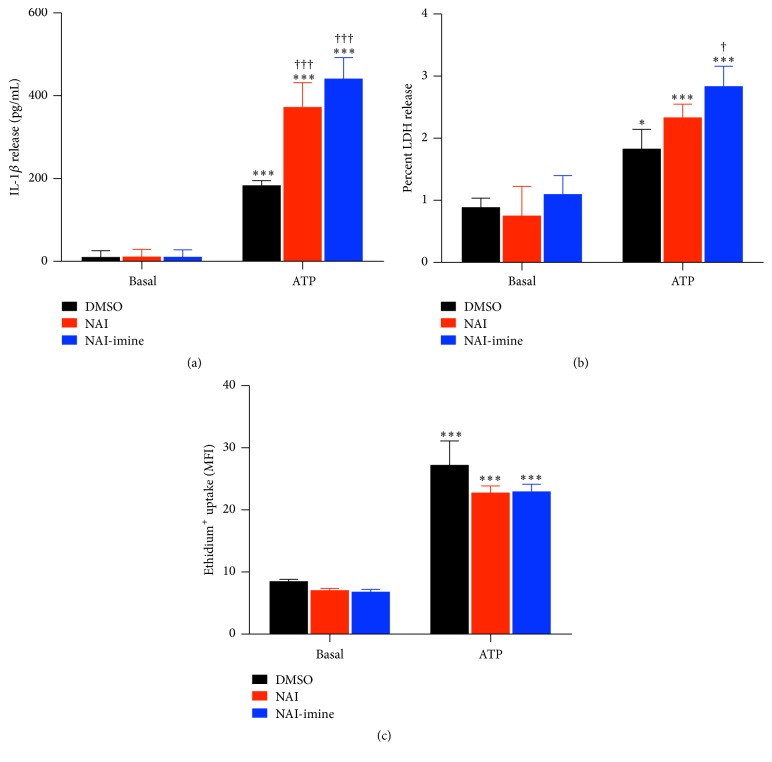
NAI or NAI-imine increase P2X7-mediated IL-1*β* release but not ethidium^+^ uptake. LPS-primed J774 cells in physiological medium were preincubated in the presence of DMSO, or 1 *μ*M NAI or 1 *μ*M NAI-imine for 30 min, and then in the absence or presence of 3 mM ATP for ((a) and (b)) 20 min or (c) 5 min in the presence of 25 *μ*M ethidium bromide. (a) The IL-1*β* concentration in cell-free supernatants was measured using an ELISA. (b) The LDH activity in cell-free supernatants and cell lysates was measured using a cytotoxicity kit, and results are expressed as a percentage of maximal LDH release from lysed cells. (c) Ethidium^+^ uptake was measured by flow cytometry and is expressed as mean fluorescence intensity (MFI). ((a) to (c)) Results are mean ± SD (*n* = 3); ^*∗*^ *P* < 0.05 or ^*∗∗∗*^ *P* < 0.001 compared with corresponding basal; ^†^ *P* < 0.05 or ^†††^ *P* < 0.001 compared with DMSO and ATP.

Both NAI and NAI-imine were originally identified as potent cytotoxic drugs [18, 19]. Therefore, to determine if either of these compounds enhanced P2X7-mediated IL-1*β* release by cytotoxicity, LPS-primed J774 cells were preincubated in the presence of DMSO, 1 *μ*M NAI, or 1 *μ*M NAI-imine prior to incubation with 3 mM ATP, and the subsequent release of IL-1*β* and LDH was measured from the same supernatants. As above (Figures 2 and 3), NAI and NAI-imine increased ATP-induced IL-1*β* release by approximately twofold (Figure 4(a)). Basal IL-1*β* release in the presence of DMSO or either isatin derivative was similar (Figure 4(a)). ATP induced a small but significant amount of LDH release from LPS-primed J774 cells compared to cells incubated with DMSO alone (Figure 4(b)). Preincubation with NAI-imine, but not NAI, induced a small but significant increase in ATP-induced LDH release (average ATP-induced LDH release of 1.74% and 1.58%, resp.) compared to cells incubated with both DMSO and ATP (average ATP-induced LDH release of 0.94%) (Figure 4(b)). Basal LDH release in the presence of DMSO or either isatin derivative was similar (Figure 4(b)).

To determine if NAI or NAI-imine enhanced P2X7-induced IL-1*β* release by increasing P2X7 activity, LPS-primed J774 cells were preincubated in the presence of DMSO, 1 *μ*M NAI, or 1 *μ*M NAI-imine before flow cytometric measurements of 3 mM ATP-induced ethidium uptake^+^. ATP induced significant ethidium uptake^+^ into cells compared to cells incubated in the absence of ATP (Figure 4(c)). Neither NAI nor NAI-imine altered ATP-induced ethidium uptake^+^ into cells (Figure 4(c)). Basal ethidium uptake^+^ in the presence of DMSO or either isatin derivative was similar (Figure 4(c)).

### 3.4. NAI and NAI-Imine Induce Cytotoxicity in a Concentration-Dependent Manner

**Figure 5:**
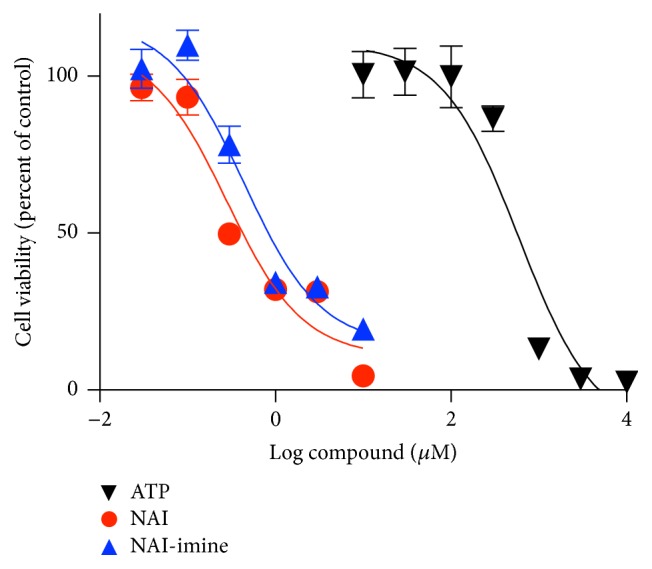
NAI and NAI-imine induces cytotoxicity in a concentration-dependent manner. LPS-primed J774 cells in complete medium were incubated with ATP, NAI, or NAI-imine (as indicated) for 24 h and then with CellTiter 96® AQ_UEOUS_ One Solution for 2 h. The viability of cells incubated with ATP or isatin derivatives was determined as percentage of the maximal absorbance of cells incubated in the absence of ATP or in the presence of DMSO, respectively. Results are mean ± SD (*n* = 3).

As stated above, NAI and NAI-imine are potent cytotoxic drugs. Therefore, to determine if these compounds induce cytotoxicity in J774 cells, LPS-primed J774 cells were incubated for 24 h in the presence of DMSO or increasing concentrations of NAI and NAI-imine, and cytotoxicity was indirectly assessed by measurements of cell viability using a tetrazolium-based assay. ATP, which induces detectable death in J774 cells in 2 to 6 h [26, 27], was used as a positive control and for comparison. As expected, ATP induced cytotoxicity in a concentration-dependent manner, with a half maximal inhibitory concentration (IC_50_) value of 596 ± 34 *μ*M and with maximal cell death induced at 3 mM ATP (Figure 5). NAI and NAI-imine also induced cytotoxicity in a concentration-dependent manner, with IC_50_ values of 279 ± 9 nM and 442 ± 105 nM, respectively, up to three orders of magnitude greater than ATP and with maximal cell death induced at 10 *μ*M for both compounds (Figure 5).

## 4. Discussion

The current study demonstrates that the* N*-alkyl-substituted isatins, NAI and NAI-imine, enhance P2X7-induced but not basal IL-1*β* release from LPS-primed murine J774 macrophages. In contrast NAI and NAI-imine did not enhance P2X7-induced dye uptake (pore formation) indicating that the effect of these compounds on ATP-induced IL-1*β* release was downstream of P2X7 activation. In contrast, others have shown that the histamine H1 receptor antagonist, clemastine, enhances both ATP-induced IL-1*β* release from human monocytes and ATP-induced dye uptake into HEK-293 cells expressing human P2X7 [28]. Similarly, the anesthetic, sevoflurane, enhances both ATP-induced caspase-1 activation (which mediates P2X7-induced IL-1*β* release in J774 macrophages [23]) and ATP-induced dye uptake into J774 macrophages [29]. Finally, the cyclooxygenase inhibitor, tenidap, and a ginsenoside metabolite of the Chinese herb, Ginseng, can also enhance ATP-induced dye uptake into J774 macrophages [30, 31], although the impact of these compounds on ATP-induced IL-1*β* release was not reported. Thus, the current study demonstrates that NAI and NAI-imine can enhance P2X7-induced IL-1*β* release in the absence of altered P2X7 activation, describing a novel mechanism by which these compounds can enhance P2X7-induced IL-1*β* release from macrophages. It remains to be determined if NAI and NAI-imine can enhance P2X7-induced IL-1*α* or IL-18 release from J774 macrophages, IL-1 family cytokines, that are also released from murine macrophages following P2X7 activation [23].

In the current study, P2X7-induced IL-1*β* release from J774 macrophages was associated with a small amount of LDH release (cytolysis). However, NAI and NAI-imine had no or minimal effect on P2X7-induced LDH release, respectively. Previous studies have demonstrated that P2X7-induced IL-1*β* release from primary murine macrophages and human monocytes can occur prior to [6] or in the absence of cell death [7], respectively. Furthermore, Vine and colleagues have described the mechanism for* N*-alkylisatin-induced cell death to be caspase-3/7 dependent in human cancer cell lines [18] indicating that these compounds mediate apoptosis rather than necrosis. Collectively, this suggests that the mechanism by which NAI and to a large extent NAI-imine enhance P2X7-induced IL-1*β* release is independent of cell rupture.

The mechanism by which NAI and NAI-imine enhance P2X7-induced IL-1*β* release remains unknown.* N*-alkyl-substituted isatins inhibit the polymerization of purified bovine neuronal tubulin and enhance microtubule fragmentation in human U937 lymphoma cells [18]. This suggests that NAI and NAI-imine may impair microtubule formation to enhance P2X7-induced IL-1*β* release from J774 macrophages. In contrast, others have shown that colchicine and nocodazole (which disrupt microtubules) and suberoylanilide hydroxamic acid and ITF2357 (which impair tubulin hyperacetylation) prevent P2X7-induced IL-1*β* release [8–10], while the microtubule stabilizing agent, paclitaxel, augments P2X7-induced IL-1*β* release [10]. Reasons for differences between these previous studies and the current study remain unknown.

The presence of the methoxybenzyl group at the N1 position of NAI and NAI-imine appears to be important for enhanced P2X7-induced IL-1*β* release. As the parent molecule, 5,7-dibromoisatin, had minimal effect on P2X7-induced IL-1*β* release. Similarly, 1*N*-methylisatin had no effect on P2X7-induced IL-1*β* release, highlighting the importance of the chain length and/or hydrophobicity at N1 for activity. Despite reports of anti-inflammatory actions of isatin and various isatin derivatives [14], the compounds tested in the current study did not impair P2X7-induced IL-1*β* release. However, it should be noted that the anti-inflammatory actions of isatin and isatin derivatives* in vitro* [32] are observed at concentrations greater than those used in the current study (30–100 *μ*M versus 1 *μ*M, resp.).

Finally, the current study demonstrates that 24-hour incubation with NAI and NAI-imine (in the absence of exogenous ATP) induces the death of LPS-primed J774 macrophages with IC_50_ values of 279 nM and 442 nM, respectively. These IC_50_ values are half that required to kill human MDA-MB-231 breast cancer and Jurkat lymphoma cell lines and one log lower for human MCF-7 breast cancer and U937 lymphoma cell lines and human colon, pancreatic, and skin cell lines, when examined using the same cytotoxicity assay [18, 19] as used in the current study. Notably, the twofold difference in the IC_50_ values between NAI and NAI-imine for J774 cells is also observed for both MDA-MB-231 and MCF-7 cells [19] suggesting that the presence of the* para*-phenylpropionic acid linker partly impairs the cytotoxic efficacy of NAI-imine compared to NAI. Nevertheless, this imine-based linker is useful for conjugation to targeting molecules to greatly increase the uptake and cytotoxic efficacy of NAI-imine [19].

The capacity of NAI and NAI-imine to enhance P2X7-induced IL-1*β* release from macrophages may have implications in cancer therapy. Various chemotherapeutic agents can cause ATP release from cancer cells [33], which in turn activates P2X7 in a paracrine manner to mediate IL-1*β* release from antigen presenting cells to promote antitumor immunity [34]. Thus, the possibility remains is that, in addition to NAI-containing drugs directly killing cancer cells, these compounds may also cause ATP release, which then act in concert to promote P2X7-induced IL-1*β* release and drive antitumor immunity. Alternatively, but not mutually exclusive to this, given that NAI and NAI-imine induce macrophage death, NAI-containing drugs may selectively kill tumor-associated macrophages, which have the capacity to promote tumorigenesis [35]. Thereby, providing an additional mechanism by which these compounds acts as anticancer agents. Finally, it should be noted that in the absence of exogenous LPS or ATP, colchicine and other microtubule destabilizers can increase IL-1*β* (and IL-1*α*) mRNA expression and protein production in human monocytes at 4 h and 18 h of incubation, respectively [36, 37]. Thus, the current and previous studies indicate time-dependent and other roles for microtubule destabilizers in IL-1 synthesis and release and that compounds such as colchicine may also mediate antitumor effects through both cytotoxicity of tumor cells and promotion of antitumor immune responses.
